# Mannan-Binding Lectin Deficiency Limits Inflammation-induced Myeloid-Derived Suppressor Cells Expansion via Modulating Tumor Necrosis Factor Alpha-triggered Apoptosis

**DOI:** 10.7150/ijbs.68865

**Published:** 2022-01-26

**Authors:** Lijun Dong, Honglian Jiang, Kai Chen, Jingwen Xie, Qing An, Fan Deng, Zhengyang Sun, Yunzhi Liu, Jia Zhou, Liyun Zhang, Xiao Lu, Mingyong Wang, Zhengliang Chen, Xiaoming Zou, Daming Zuo

**Affiliations:** 1 The Fifth Affiliated Hospital, Southern Medical University, Guangzhou, Guangdong 510900, China; 2 Department of Medical Laboratory, School of Laboratory Medicine and Biotechnology, Southern Medical University, Guangzhou, Guangdong 510515, China; 3 Department of Immunology, School of Basic Medical Sciences, Southern Medical University, Guangzhou, Guangdong 510515, China; 4 State Key Laboratory of Organ Failure Research, Department of Cardiology, Nanfang Hospital, Southern Medical University, 510515, Guangzhou, China; 5 School of Laboratory Medicine, Xinxiang Medical University, Xinxiang 453003, China; 6 Guangdong Provincial Key Laboratory of Proteomics, Southern Medical University, Guangzhou, Guangdong 510515, China; 7 Microbiome Medicine Center, Zhujiang Hospital, Southern Medical University, Guangzhou, Guangdong 510282, China

**Keywords:** mannan-binding lectin, myeloid-derived suppressor cells, tumor necrosis factor-α, apoptosis

## Abstract

**Background:** Mannan-binding lectin (MBL), a soluble pattern recognition molecule in the innate immune system, is reported to be associated with the function of immune cells. Myeloid-derived suppressor cells (MDSCs) are mainly characterized by immunosuppressive activities involving several inflammatory diseases such as cancer, infection, and arthritis. Some of the factors inducing their apoptosis are known, however, mechanisms have not been identified. The underlying impact of MBL on the MDSCs especially under inflammatory conditions remains unknown. This study was designed to investigate whether MBL affects MDSCs survival during inflammation conditions.

**Methods:** WT and MBL-deficient (MBL^-/-^) mice were induced on day 0 of the experiment by subcutaneous injection of complete Freund's adjuvant and then injected with incomplete Freund's adjuvant into the knee joint space under general anesthesia on day 14 to induce inflammatory arthritis. The proportions of MDSCs in the spleen and blood and the serum level of the inflammatory cytokines were measured. In vitro study, MDSCs were isolated from the bone marrow of WT and MBL^-/-^ mice and cultured in the presence of interleukin-6 (IL-6) and granulocyte-macrophage colony-stimulating factor (GM-CSF) for 5 days with or without tumor necrosis factor-alpha (TNF-α).

**Results:** After adjuvant treatment, MBL^-/-^ mice had a significantly lower frequency of MDSCs as well as elevated serum inflammatory cytokines levels compared to WT mice. MBL deficiency markedly inhibited the MDSCs frequency from mice bone marrow induced by IL-6 and GM-CSF in the presence of TNF-α *in vitro*. Mechanistic studies established that MBL inhibited MDSCs apoptosis via down-regulation of TNF-α/tumor necrosis factor-alpha receptor 1 (TNFR1) signaling pathway and subsequent caspase 3-dependent manner.

**Conclusion:** Mannan-binding lectin deficiency inhibits myeloid-derived suppressor cells expansion via modulating TNF-α triggered apoptosis.

## Introduction

MDSCs are immature myeloid cells with immunosuppressive functions. In mice, MDSCs are characterized by the expression of cell surface markers CD11b and Gr-1 and are further defined into CD11b^+^ly6C^high^ ly6G^low^ or CD11b^+^ly6C^low^ly6G^high^ subset 1. MDSCs are potent suppressors of various T-cell functions characterized by the increased production of reactive oxygen and nitrogen species, and of arginase 1 (arg 1). Accumulating evidence has shown that MDSCs regulate immune responses during inflammation-related diseases, especially rheumatoid arthritis (RA). In the mouse collagen-induced arthritis model, some recent papers suggest MDSCs play an immune-suppressive role 2. Nevertheless, a previous study has shown that MDSCs can also have a pro-inflammatory role and promote the RA severity in mice by sustaining Th17 cell differentiation 3.

Mannan-binding lectin (MBL), also called mannose-binding lectin or mannan-binding protein (MBP), is primarily synthesized in the liver and is also expressed in some myeloid cells 4. Emerging evidence suggested that MBL deficiency is associated with susceptibility to several diseases such as gastric cancer 5, infection 6, inflammatory bowel disease 7, and systemic lupus erythematosus 8. As an important part of the immune system, the most prominent role of MBL is its impact on a variety of immune cells 9-11. It is noteworthy to mention that we previously observed MBL could markedly inhibit the osteoclast formation from human blood monocytes induced by receptor activator of nuclear factor-κB ligand (RANKL) and macrophage colony-stimulating factor in vitro 12. Besides, MBL inhibited IL-2-induced signal transducers and activators of transcription 5 (STAT5) activation in NK cells 13. MBL also markedly inhibited T-cell proliferation induced by anti-CD3 and anti-CD28 antibodies 9. Therefore, it is possible that MBL might influence the differentiation of MDSCs, which, however, so far has no evidence to support it.

In this study, we used MBL^-/-^ mice to generate inflammatory arthritis for evaluating the function of MBL on the process of inflammation, especially the survival and function of MDSCs. The results showed that MBL^-/-^ mice exhibited more severe inflammation and a lower frequency of MDSCs. It is noteworthy that we demonstrated that the frequency of total MDSCs, G-MDSCs, and M-MDSCs derived from MBL^-/-^ mice was lower than that from WT mice. Moreover, MBL deficiency reduces the apoptosis of MDSCs mediated by TNF-α/TNFR1, which depends on the FADD/TRADD/caspase3/8 signaling pathway. In summary, our findings provide the first line of evidence that the function of MBL in MDSCs survival in the presence of TNF-α, indicating the association of MBL with MDSCs-related disease, especially in conditions with high TNF-α levels.

## Materials and Methods

### Experimental Animals

Male C57BL/6J mice (8 weeks old, 21-25 g) were purchased from the Laboratory Animal Center of Southern Medical University (Guangzhou, China). MBL^-/-^ C57BL/6J (8 weeks old, 21-25 g) mice were obtained from the Jackson Laboratory (Bar Harbor, ME, USA). The mice were housed in a specific pathogen-free condition, on a 12-h light-dark cycle, and with food and water ad libitum. All animal experiments in this study were approved by the Welfare and Ethical Committee for Experimental Animal Care of Southern Medical University.

### Reagents

Complete Freund's adjuvant (CFA) (4 mg/ml heat-killed Mycobacterium tuberculosis) and incomplete Freund's adjuvant (IFA) were obtained from Chondrex (Washington, USA). Recombinant mouse IL-6 (216-16), GM-CSF (500-P65), and TNF-α (315-01A) were purchased from Peprotech (London, UK). Anti-caspase 3 antibody (19677-1-AP), anti-caspase 8 antibody (13423-1-AP), anti-FADD antibody (14906-1-AP), anti-TRADD antibody (15468-1-AP), and anti-β-actin antibody (66009-1-Ig) were purchased from Proteintech (Chicago, USA). Anti-Gr-1 antibody (RB6-8C5), Anti-CD11b antibody (M1/70), Anti-ly6G antibody (1A8), Anti-ly6C antibody (ER-MP20), and cell apoptosis kit (V13241) were purchased from Thermo Fisher (Waltham, USA). IL-10 (88-7105-76), IL-1β (88-7013-22), IL-6 (BMS603-2), IFN-γ (88-7314-22) mouse ELISA kits were purchased from Thermo Fisher (Waltham, USA). Trizol, TranScript Allin-One First-Strand cDNA Synthesis SuperMix (AT341), and TransStart Tip Green qPCR SuperMix (AQ141) were purchased from TransGen (Beijing, China).

### Induction of adjuvant-induced arthritis (AIA)

Adjuvant arthritis was induced on day 0 of the experiment by bilateral axillary subcutaneous injection of 0.1ml of complete Freund's adjuvant (CFA) (4 mg/ml heat-killed mycobacterium tuberculosis). Mice (8 weeks old, 22-25 g) were injected with 20 μl of incomplete Freund's adjuvant (IFA) into the bilateral knee joint space under general anesthesia on day 14 according to the previous study 14. To block TNF-α, mice were injected infliximab (10 μg/g, i.p.) after receiving the first immunization.

### Histological evaluation in AIA

The histopathological score was scored as described previously 15. Scores were as following: For inflammation score 0: No inflammation; score 1: Slight thickening of lining layer or some infiltrating cells in sublining layer; score 2: Slight thickening of lining layer plus some infiltrating cells in sublining layer; score 3: Thickening of lining layer, influx of cells in sublining layer and presence of cells in the synovial space and score 4: Synovium highly infiltrated with many inflammatory cells. For cartilage erosion score 0: No destruction; score 1: Minimal erosion limited to single spots; score 2: Slight to moderate erosion in a limited area; score 3: More extended erosions and score 4: General destruction.

### MDSCs Differentiation Assay

MDSCs were isolated from the bone marrow of WT and MBL^-/-^ mice and cultured in the presence of 10 ng/ml GM-CSF (PeproTech, Cranbury, NJ, USA) and 10 ng/ml of IL-6 (PeproTech, Cranbury, NJ, USA) for 5 days. In some experiments, 5 ng/ml of TNF-α (PeproTech, Cranbury, NJ, USA) were added to the cells, which were incubated with GM-CSF. After the different incubation periods, cell phenotypes were determined by flow cytometry analysis.

### Immunosuppression assay

Carboxylfluorescein succinimidyl ester (CFSE) labeling was used to detect the proliferation of CD3+ T cells. Briefly, 1.5×10^6^ CD3^+^ T cells from WT and MBL^-/-^ were co-cultured with MDSCs at ratios of 1:0, 1:1, and 4:1 in RPMI-1640 medium, supplemented with 10% FBS and stimulated with anti-CD3 and CD28 monoclonal antibodies. All CD3^+^ T cells were seeded into a 96-well plate in the presence or absence of MDSCs, as mentioned above. The cells were harvested following culturing for 5 days. As the CFSE signal was diluted with each cell division, cells exhibiting low fluorescence intensity of CFSE were considered to have proliferated.

### RNA Isolation and Quantitative Real-time PCR

Total RNA was extracted using Trizol reagent according to the manufacturer's instructions. First-strand complementary DNA (cDNA) was obtained using the PrimeaScript™ II First-Strand cDNA synthesis kit, according to the manufacturer's instructions. Subsequently, SYBR green-based quantitative real-time PCR (qPCR) was performed using a power SYBR green master mix and 7500 Fast Real-Time PCR system (Applied Biosystems). The GAPDH gene was used as an internal control. The sequences of the primers used for PCR are listed in **Table 1**.

### ELISA assay

Mice blood samples and cell culture supernatant were centrifuged at 8,000×g for 15 minutes, and then the supernatant was collected and set aside at -80^◦^C for serum cytokine analysis. Cytokine levels in the sera were assessed using commercial ELISA kits.

### Immunoprecipitation and Immunoblotting

MDSCs were harvested in RIPA buffer with protease inhibitor tablets and sonicated on ice. The lysate was then centrifuged at 14,000 rpm at 4°C for 15 minutes, and the supernatant was reserved. Protein lysates were denatured in the SDS sample buffer at 100 °C for 5 minutes and then were separated by 10% SDS-PAGE and transferred onto polyvinylidene fluoride membranes (Millipore, Bedford, USA). After blocking in 5% milk, the membrane was incubated with a primary antibody at 4°C for overnight. After washing, the membranes were incubated with horseradish peroxidase (HRP)-conjugated secondary antibodies at room temperature for 1 hour. Detection and analysis were performed using the Chemidoc XRS Image system and Image Lab 5.0 software (BioRad). For immunoprecipitation, whole-cell lysates were incubated with 1 μg of antibody and protein A/G agarose (Santa Cruz Biotechnology, Santa Cruz, CA, USA) at 4 °C overnight. The eluted immunoprecipitates were resolved via SDS-PAGE, and the associations between proteins of interest were examined using specific antibodies.

### Statistical analysis

All results were expressed as mean ± SEM. One-way ANOVA was used for comparisons among multiple groups. Differences between the two groups in the experiments were analyzed by Student's t-test. p<0.05 was considered statistically significant.

## Results

### MBL deficiency exacerbates AIA in mice

**Figure 1:**
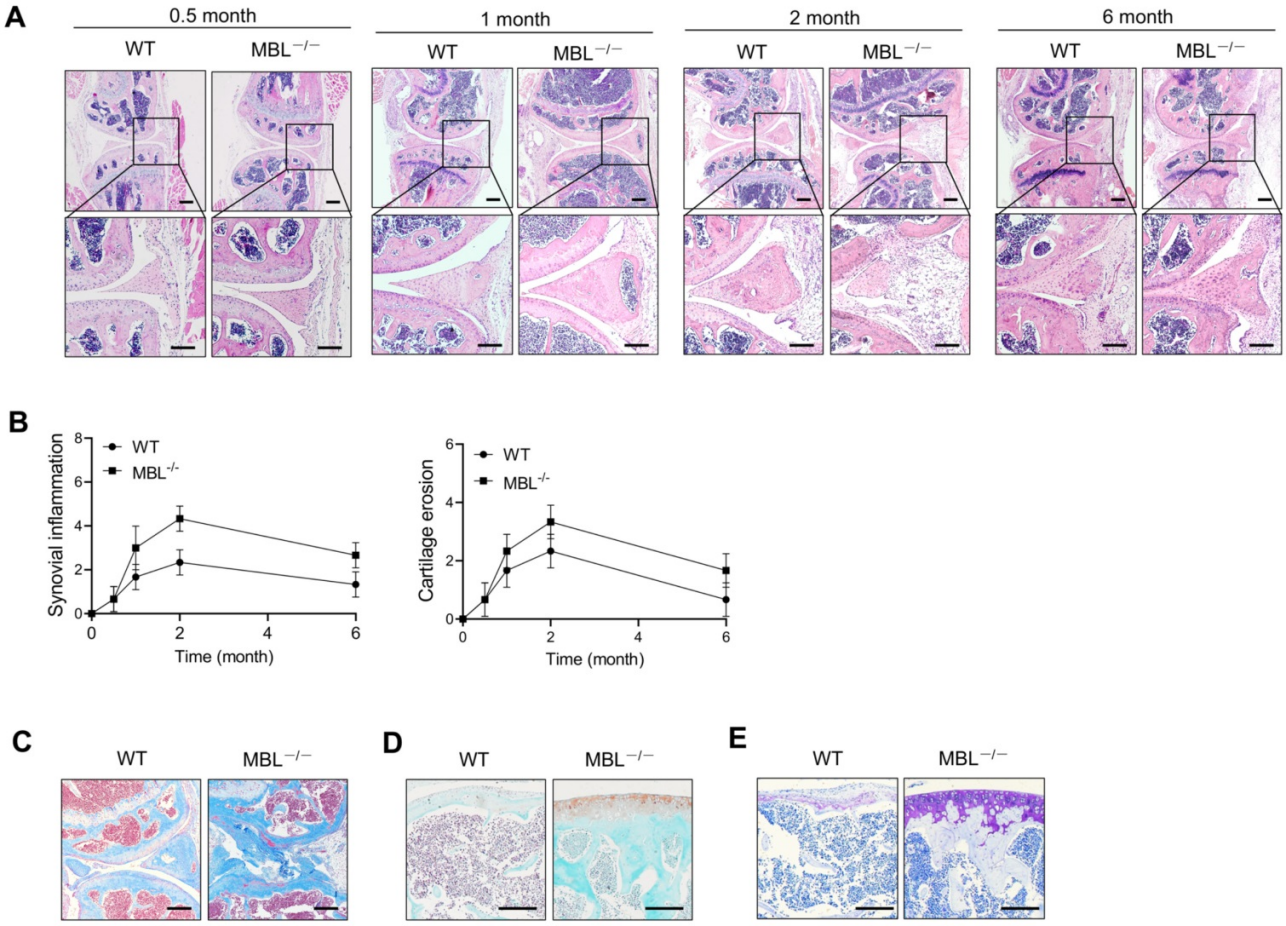
** MBL ablation renders mice more susceptible to AIA.** WT and MBL^-/-^ mice (n=6, 8 weeks old) arthritis were induced by immunization with Freund's complete adjuvant and Freund's incomplete adjuvant. (A) Histopathological evaluation of the arthritis-induced damage to the knee joints was performed with H&E staining. Bottom panels showed the higher-magnification views of the box area. Scale bars = 200 μm. (B) Histological scores of inflammation and cartilage erosion from the experiment shown. (C-E) Bone destruction and cartilage damage in knee bone from WT and MBL-/- mice were determined after 2 months with adjuvant induction. Masson's trichrome staining was used to assess collagen deposition (C). Cartilage erosion was assessed by safranin O-fast green staining (D). Toluidine blue staining was used to qualitatively assess the proteoglycan content in the cartilage (E). Scale bar = 50 μm. Data are representative of three independent experiments with similar results.

To define the function of MBL in adjuvant-induced arthritis (AIA) of mice, sex and age-matched C57BL/6 WT and MBL^-/-^ mice were received adjuvant immunization. The data showed that adjuvant-treated MBL^-/-^ mice displayed severe joint destruction compared with WT counterparts, especially at 2 months after treatment with incomplete Freund's adjuvant **(Fig. 1A)**. When evaluated with histopathological parameters, MBL^-/-^ AIA mice showed high scores in both synovial inflammation and cartilage erosion, compared to WT counterpart mice **(Fig. 1B)**. Besides, MBL^-/-^ mice exhibited more osteoporosis, collagen deposition, and damage of cartilage than WT mice after immunization with adjuvant **(Fig. 1C-E)**. Together, these results indicate a potential protective role of MBL in the pathogenesis of experimental AIA.

### MBL deficiency inhibits MDSCs accumulation of mouse AIA

**Figure 2:**
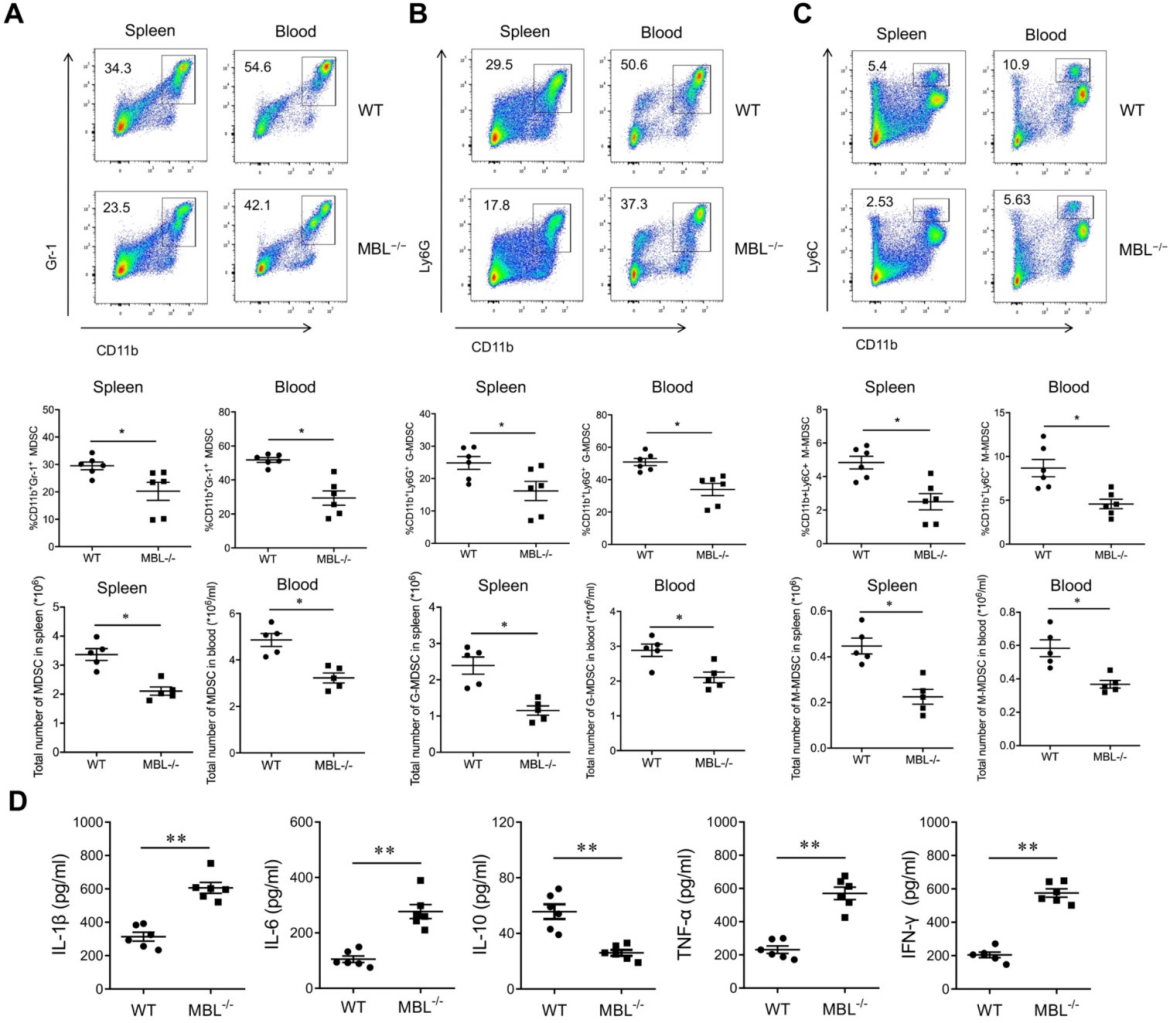
** MDSC is decreased in MBL-deficient AIA mice.** WT and MBL^-/-^ mice (n=6, 8 weeks old) arthritis were induced by immunization with Freund's complete adjuvant and Freund's incomplete adjuvant. The frequency and the count of (A) MDSCs (CD11b^+^Gr1^+^), (B) G-MDSCs (CD11b^+^ly6G^+^), and (C) M-MDSCs (CD11b^+^ly6C^+^) in spleen and blood were analyzed by flow cytometry at 2 months after Freund's complete adjuvant injection. (D) 2 months after the primary immunization, the serum levels of IL-1β, IL-6, IL-10, TNF-α, and IFN-γ from the mice was measured by ELISA. *p<0.05, **p<0.01. Data are representative of three independent experiments with similar results.

To explore the role of MBL in inflammation of AIA mice, sex and age-matched C57BL/6 WT and MBL^-/-^ mice were received adjuvant immunization, followed by the analysis of cytokines levels as well as MDSCs frequency. As shown, adjuvant-treated MBL^-/-^ mice displayed a marked decrease in the frequency of MDSCs in the spleen and blood compared with WT counterparts **(Fig. 2A)**. Also, G-MDSCs **(Fig. 2B)** and M-MDSCs **(Fig. 2C)** showed the same trend in both spleen and blood. Serum collected from MBL^-/-^ mice showed significantly increased levels of pro-inflammatory cytokines (IL-1β, IL-6, TNF-α, and IFN-γ) and decreased levels of anti-inflammatory cytokine (IL-10) compared with that from WT mice after adjuvant immunization. **(Fig. 2D)**. Collectively, these results indicated a potential role of MBL in MDSCs function of inflammatory arthritis.

### MBL does not affect MDSCs frequency under physiological conditions

**Figure 3:**
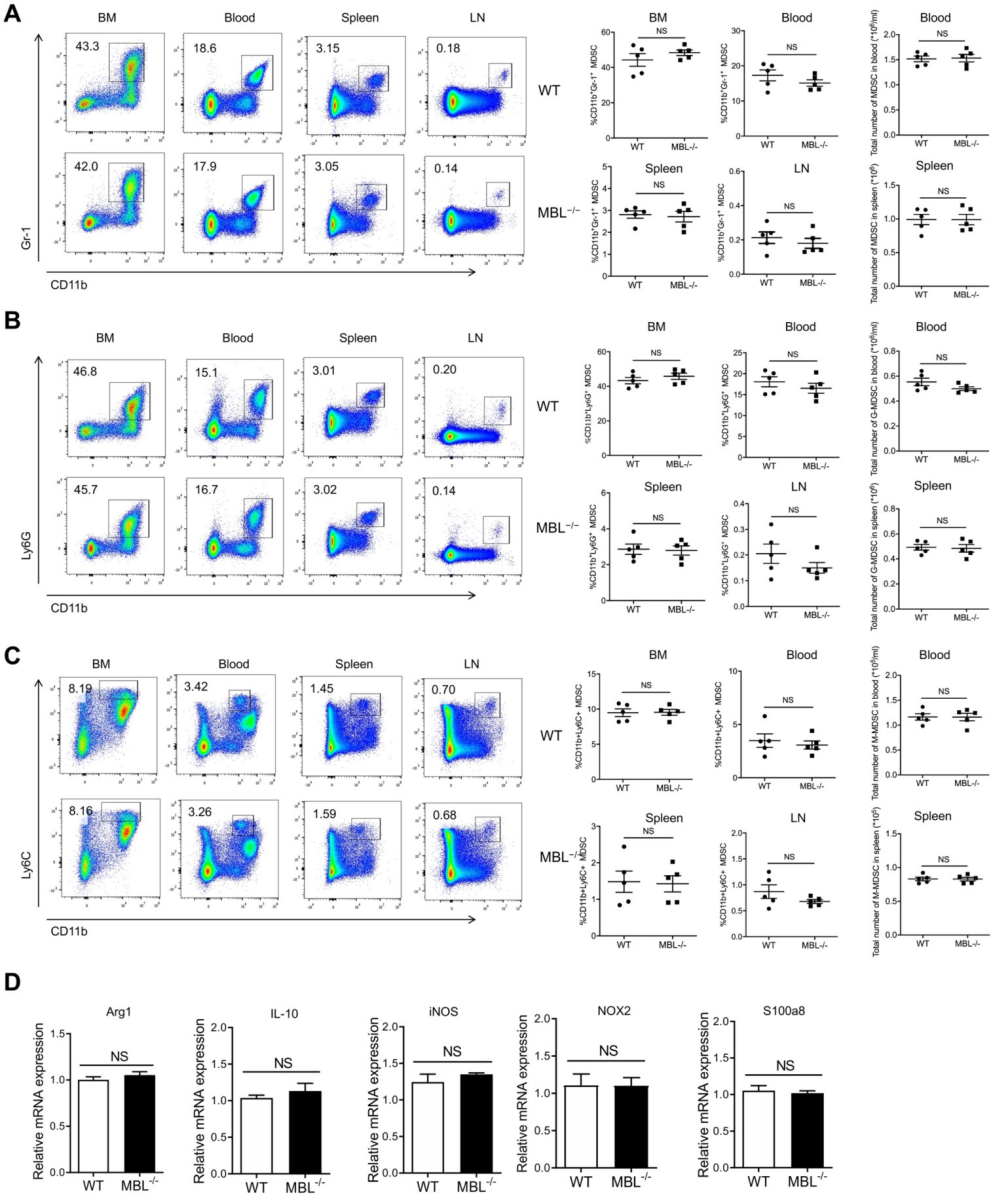
** MBL does not involve in MDSCs expansion in vivo under a physiological state.** The frequency of (A) MDSCs, (B) G-MDSCs, and (C) M-MDSCs in bone marrow, blood, spleen, and lymph node of 8-week-old WT and MBL^-/-^ mice (n=5 per group) were analyzed by flow cytometry. (D) MDSCs were isolated from the spleen of WT and MBL^-/-^ mice using magnetic beads conjugated to anti-Gr1 antibody. The mRNA expression of arg1, IL-10, iNOS, NOX2, and s100a8 in MDSCs was analyzed by qRT-PCR. NS, not significant. Data from one representative experiment of three independent experiments are presented.

The above results indicated that MBL^-/-^ mice show less frequency of MDSCs compared with WT mice, so we speculated that MBL might be involved in the differentiation or survival of MDSCs. To verify this conjecture, we explore MDSC subsets of bone marrow (BM), blood, spleen, and lymph nodes (LN) in MBL^-/-^ and paired WT mice under physiological conditions. Surprisingly, flow cytometry analysis showed that the percentages of CD11b^+^Gr-1^+^ MDSCs of MBL^-/-^ mice were similar with WT mice under physiological conditions **(Fig. 3A)**. Similarly, the rates of G-MDSCs **(Fig. 3B)** and M-MDSCs **(Fig. 3C)** were similar between WT and MBL^-/-^ mice. Moreover, the mRNA expression levels of several inflammatory mediator genes, including Arg1, IL-10, inducible nitric oxide synthase (iNOS), NADPH oxidase 2 (NOX2), and s100a8, were comparable in MDSCs isolated from WT and MBL^-/-^ mice **(Fig. 3D)**.

**Figure 4:**
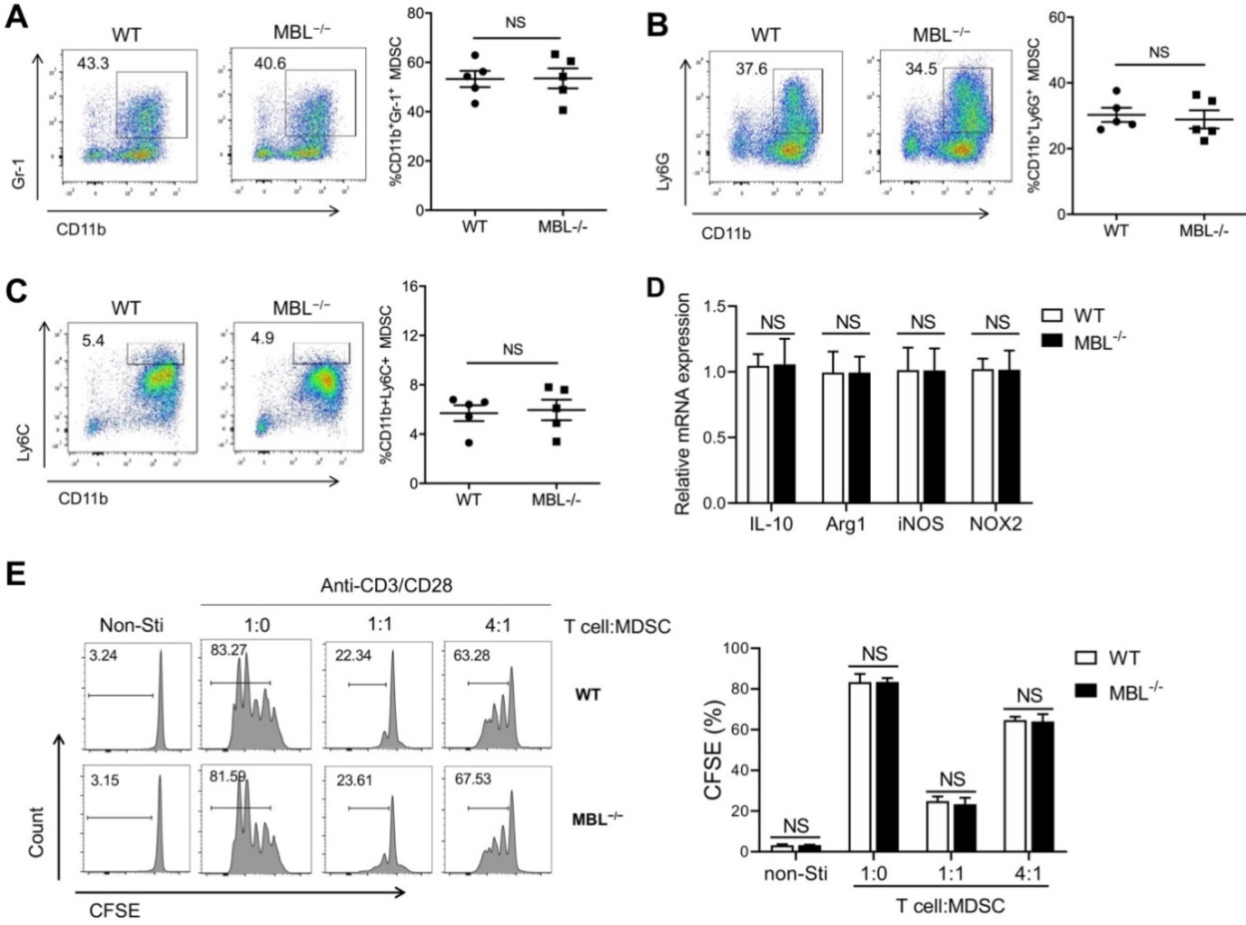
** MBL does not involve in MDSCs expansion in vitro.** Bone marrow cells from WT and MBL^-/-^ mice (8 weeks old) were treated with 10 ng/ml of GM-CSF and 10 ng/ml of IL-6 for five days. The frequency of (A) MDSCs, (B) G-MDSCs, and (C) M-MDSCs in WT and MBL^-/-^ mice (n=5 per group) were analyzed by flow cytometry. (D) The mRNA expression of Arg1, IL-10, iNOS, NOX2, and s100a8 in MDSCs was analyzed by RT-PCR. (E) MACS-purified T cells were stimulated with anti-CD3/CD28 antibodies in the presence of WT or MBL^-/-^ MDSCs. The proliferation of T cells was analyzed by flow cytometry. NS: no statistical difference, *p<0.05, **p<0.01. Data from one representative experiment of three independent experiments are presented.

Next, we investigated whether MBL affected MDSCs expansion *in vitro*. Bone marrow cells from WT and MBL^-/-^ mice were treated with 10 ng/ml of GM-CSF and 10 ng/ml of IL-6, which is known to induce MDSCs differentiation 16. As shown in **Figure 4A**, MBL deficiency did not affect MDSCs differentiation *in vitro*. In addition, the frequency of G-MDSCs **(Fig. 4B)** and M-MDSCs **(Fig. 4C)** was similar between WT and MBL^-/-^ mice. The mRNA expression of Arg1, IL-10, iNOS, and NOX2 was similar in MDSCs of WT and MBL^-/-^ mice **(Fig. 4D)**. MDSCs derived from the bone marrow of WT and MBL^-/-^ mice showed no difference in their inhibitory effects on T cells proliferation **(Fig. 4E)**. These results suggested that MBL did not influence the proportion and immunosuppression of T cell expansion of MDSCs under physiological conditions both *in vivo* and *in vitro*.

### MBL does not affect MDSCs accumulation in AIA mice after TNF-α neutralization

**Figure 5:**
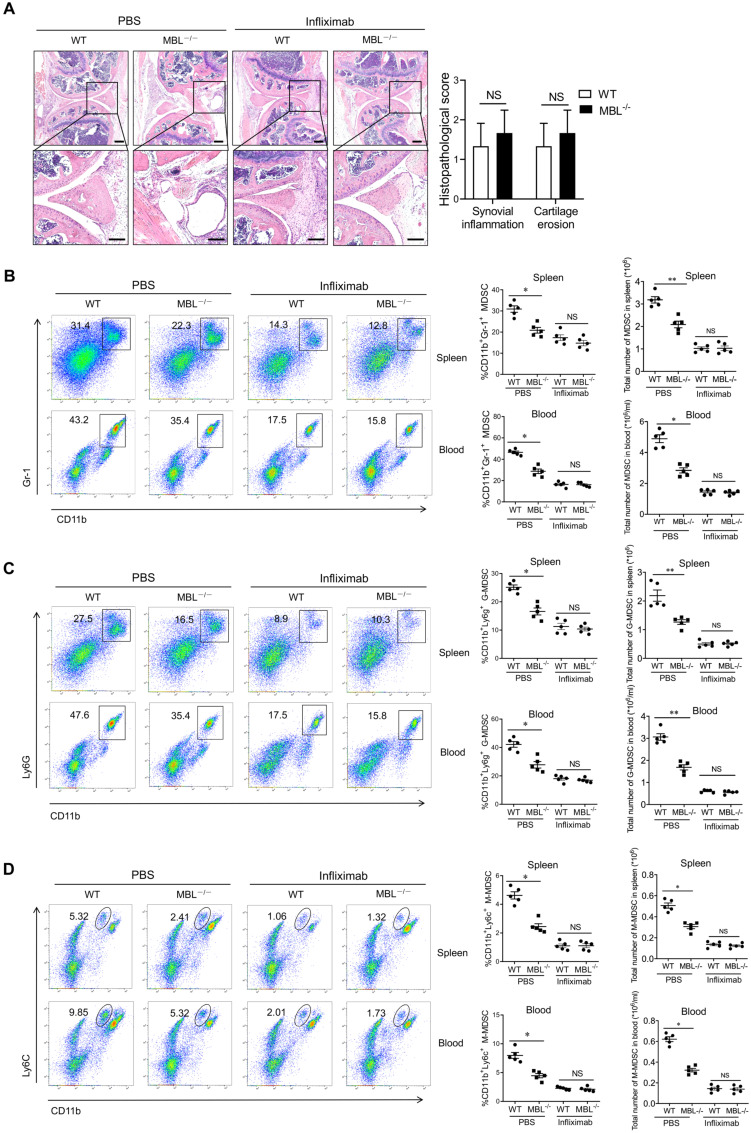
** MBL-mediated MDSCs frequency of arthritis mice is dependent on TNF-α.** The mice (n=5, 8 weeks old) were treated with Freund's complete adjuvant and Freund's incomplete adjuvant in the presence of infliximab (10 μg/g). (A) Histopathological evaluation of the arthritis-induced damage to the knee joints was performed with H&E staining. Bottom panels showed the higher-magnification views of the box area. Scale bars=200 μm. Histological scores of inflammation and cartilage erosion from the experiment shown. The frequency of (B) MDSCs (CD11b^+^Gr1^+^), (C) G-MDSCs (CD11b^+^ly6G^+^), and (D) M-MDSCs (CD11b^+^ly6C^+^) in spleen and blood were analyzed by flow cytometry at 2 months after Freund's complete adjuvant injection. *p<0.05; NS, not significant.

MBL deficiency didn't affect MDSCs frequency under physiological conditions, but increased the proportion of MDSC under pathological arthritis conditions. We, therefore, raised the question of how MBL influenced MDSCs under arthritis. Arthritis is an autoimmune disease characterized by chronic, low-grade inflammation, accompanied by increased inflammatory cytokines (i.e., IL-1β, TNF-α, and IL-6). Among those cytokines, the TNF-α signaling pathway plays a critical role in arthritis development and it serves as a therapeutic target in arthritis 17. To further validate the role of TNF-α in MBL-mediated regulation of MDSCs differentiation, mice were treated with specific TNF-α antagonist infliximab after the first immunization. Upon infliximab treatment, the knee joint injury in WT and MBL^-/-^ arthritis mice had no difference **(Fig. 5A)**. In addition, the frequency of MDSCs **(Fig. 5B)**, G-MDSCs **(Fig. 5C)**, and M-MDSCs **(Fig. 5D)** were similar between WT and MBL^-/-^ arthritis mice after blocking TNF-α. These results indicate that TNF-α is required for MBL-mediated regulation of arthritis progression and accompanying changes in MDSCs.

### MBL deficiency decreases MDSCs differentiation *in vitro* upon TNF-α stimulation

**Figure 6:**
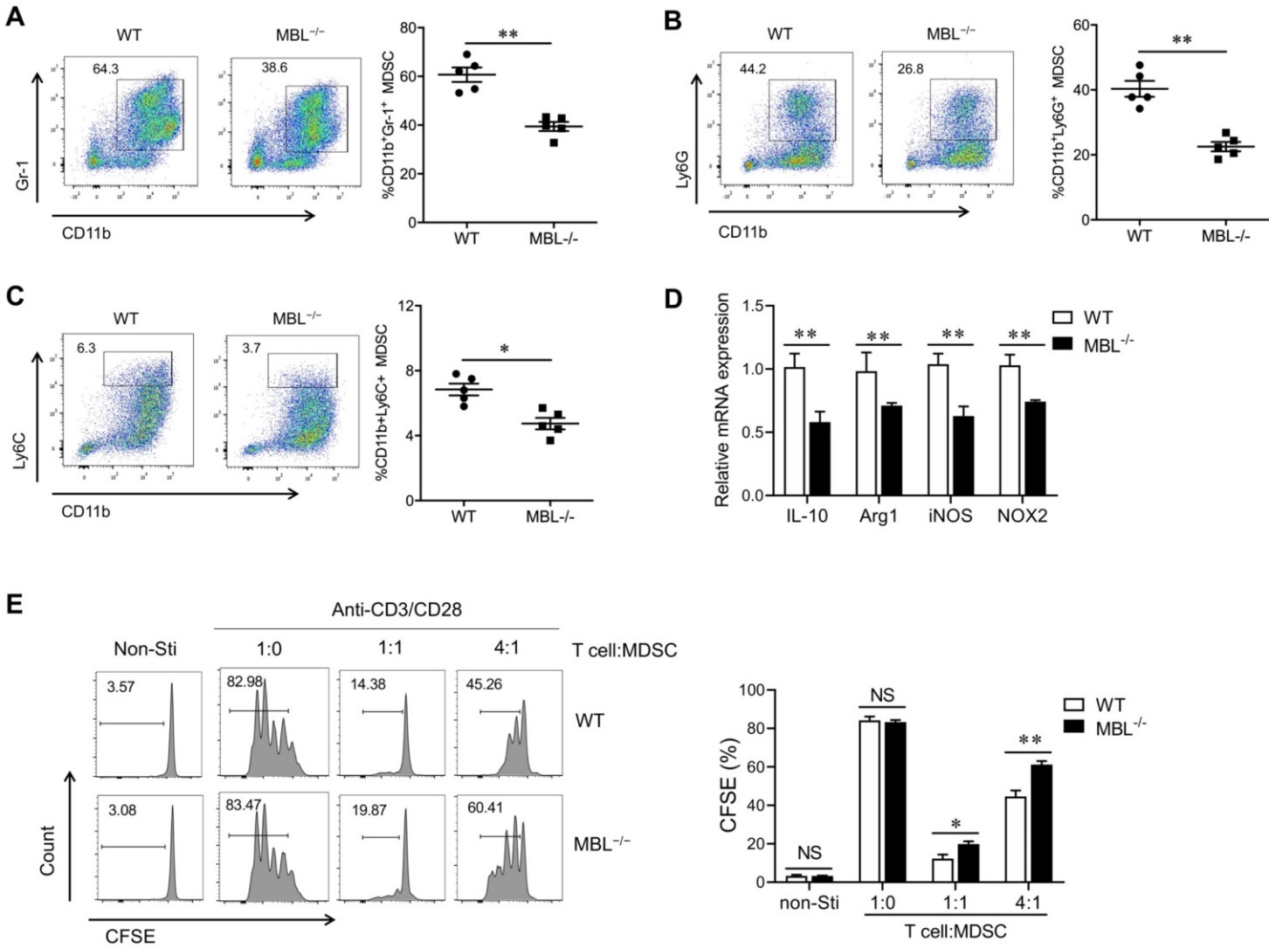
** MBL deficiency decreases MDSCs expansion in vitro in the presence of TNF-α.** Bone marrow cells from WT and MBL^-/-^ mice (8 weeks old) were treated with 10 ng/ml of GM-CSF and 10 ng/ml of IL-6 in the presence of TNF-α (5 ng/ml) for five days. The frequency of (A) MDSCs, (B) G-MDSCs, and (C) M-MDSCs in BM, blood, spleen, and LNs of 8-week-old WT and MBL^-/-^ mice (n=5 per group) were analyzed by flow cytometry. (D) The mRNA expression of arg1, IL-10, iNOS, NOX2, and s100a8 in MDSCs was analyzed by RT-PCR. (E) MACS-purified T cells were cultured with anti-CD3/CD28 antibodies in the presence of TNF-α-stimulated WT or MBL^-/-^ MDSCs for 24 hours. The proliferation of T cells was analyzed by flow cytometry. NS: no statistical difference, *p<0.05, **p<0.01. Data from one representative experiment of three independent experiments are presented.

It has been reported that TNF-α is involved in MDSC accumulation during chronic inflammation. A significantly increased plasma TNF-α level was also observed in MBL^-/-^ mice with arthritis compared with WT mice. To determine whether MBL affects MDSCs expansion under inflammatory conditions, MDSCs were differentiated from murine bone marrow cells in the presence or absence of TNF-α. Compared to MDSCs derived from WT mice, the differentiation of MBL^-/-^ MDSCs was limited in the presence of TNF-α **(Fig. 6A)**, and this effect of TNF-α was dose-dependent (**Supplementary Fig. 1**). Similar results were observed in G-MDSCs **(Fig. 6B)** and M-MDSCs **(Fig. 6C)**. MBL deficiency also decreased the mRNA expression of Arg1, IL-10, iNOS, and NOX2 of MDSCs **(Fig. 6D)**. Besides, the inhibitory function of TNF-α-stimulated MBL^-/-^ MDSCs cells on T cells is weaker than that of WT mice **(Fig. 6E)**. Thus, these results indicated that MBL deficiency suppresses MDSCs differentiation upon TNF-α stimulation.

### MBL inhibits MDSCs apoptosis in the presence of TNF-α

**Figure 7:**
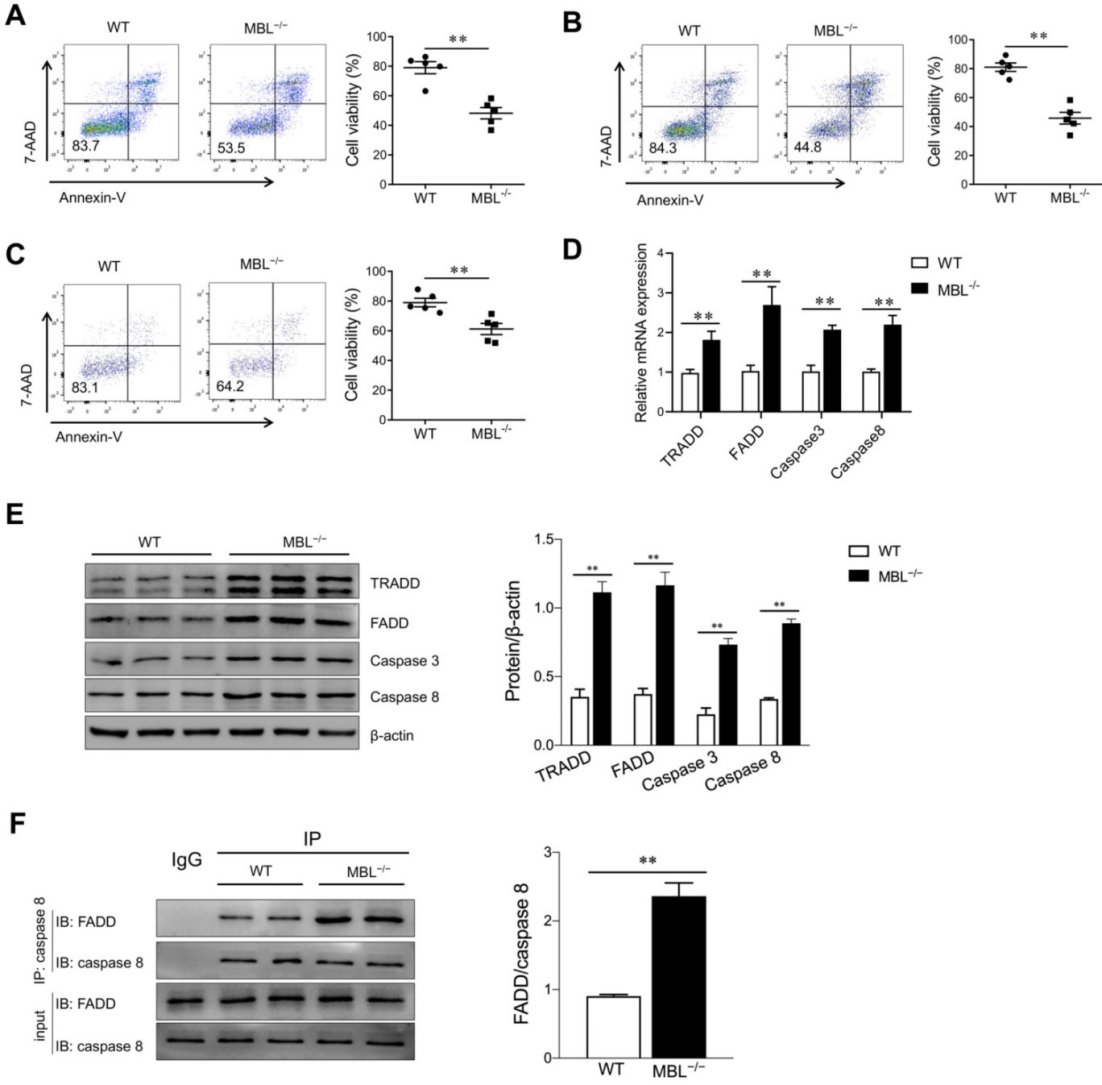
** MBL deficiency increases MDSCs apoptosis in vitro upon TNF-α treatment.** Bone marrow cells from WT and MBL^-/-^ mice (8 weeks old) were treated with 10 ng/ml of GM-CSF and 10 ng/ml of IL-6 in the presence of TNF-α (5 ng/ml) for five days. Cell viability of (A) MDSCs, (B) G-MDSCs, and (C) M-MDSCs derived from WT and MBL^-/-^ mice (n=5 per group) were analyzed by flow cytometry. (D) The mRNA and (E) protein expression of FADD, TRADD, caspase 3, and caspase 8 in MDSCs derived from WT and MBL^-/-^ mice. *p<0.05. (F) The association of caspase 8 with FADD was determined by immunoprecipitation. Data from one representative experiment of three independent experiments are presented.

Since TNF-α plays a pivotal role in cell apoptosis, we investigate whether MBL affects TNF-α-related apoptosis in MDSCs. FACS analysis revealed that MBL deficiency decreased the cell viability of MDSCs in the presence of TNF-α **(Fig. 7A)**. A lower frequency of living G-MDSCs **(Fig. 7B)** and M-MDSCs **(Fig. 7C)** derived from MBL^-/-^ mice was observed than that from WT controls. Additionally, a massive increase in mRNA levels of apoptosis-related genes (FADD, TRADD, caspase 3, and caspase 8) was exhibited in MDSCs derived from MBL^-/-^ mice compared with those from WT mice **(Fig. 7D)**. Also, western blot analysis further confirmed the increased protein level of apoptosis-related molecules of MDSCs in the MBL^-/-^ group compared to the WT group **(Fig. 7E)**. Caspase-8 is a proapoptotic member of the caspase family, activated upon stimulation of a death receptor (e.g., Fas) and recruitment of the adaptor molecule FADD. It is worth noting that immunoprecipitation results showed increased interaction between FADD and caspase 8 of MDSCs in the MBL^-/-^ group compared to the WT group **(Fig. 7F)**. According to these results, we found that MBL affected MDSCs apoptosis upon TNF-α stimulation.

### MBL maintains MDSCs survival partially depending on TNF-α/TNFR1

**Figure 8:**
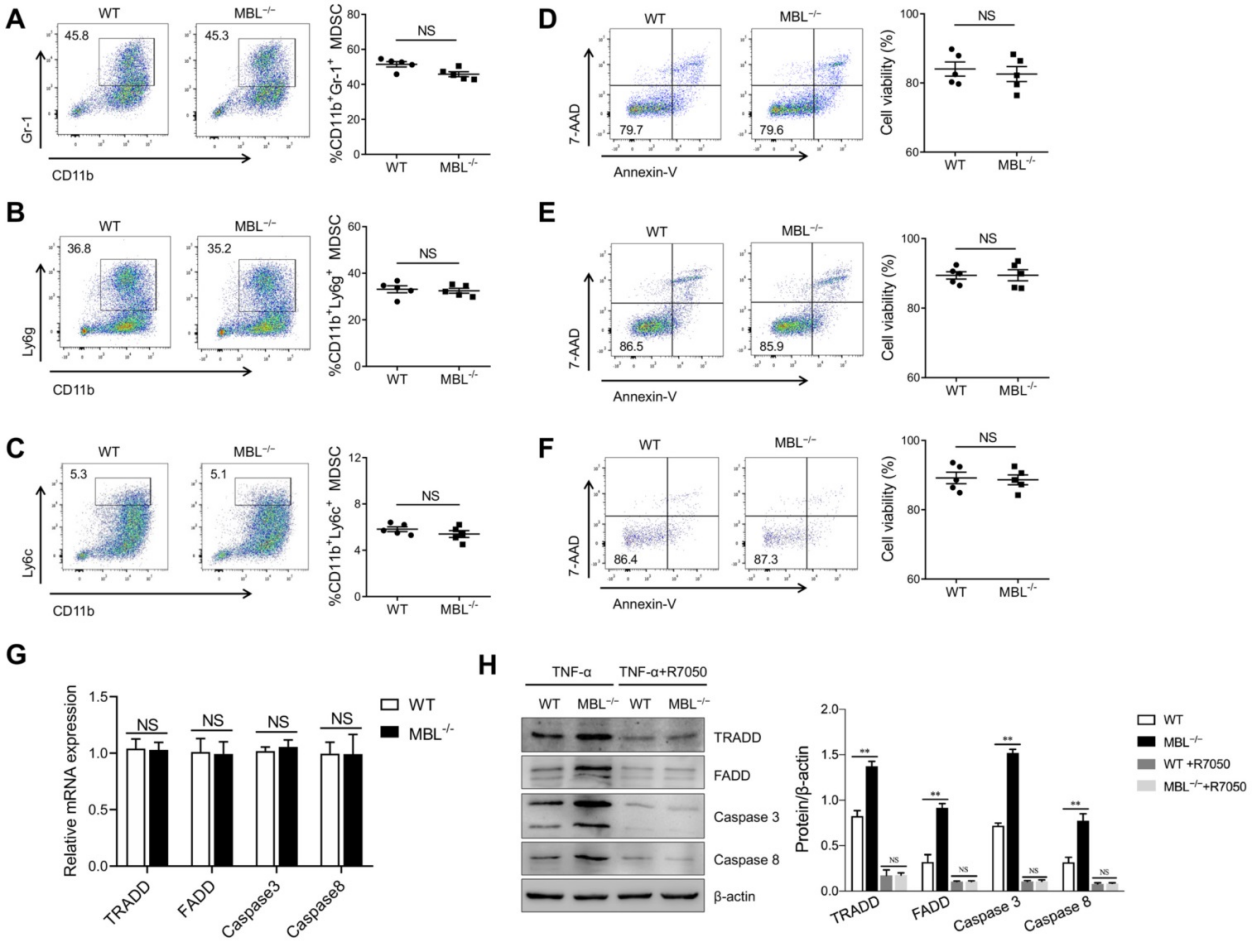
** MBL participates in MDSCs survival via the TNF-α/TNFR1 pathway.** Bone marrow cells from WT and MBL^-/-^ mice (8 weeks old) were treated with GM-CSF (10 ng/ml), IL-6 (10 ng/ml), and TNF-α (5 ng/ml) for five days with or without R-7050. The frequency of (A) MDSCs, (B) G-MDSCs, and (C) M-MDSCs in WT and MBL^-/-^ mice (n=5 per group) was analyzed by flow cytometry. Cell viability of (D) MDSCs (E) G-MDSCs and (F) M-MDSCs derived from WT and MBL^-/-^ mice (n=5 per group) were analyzed by flow cytometry. (G) The mRNA and (H) protein expression of FADD, TRADD, caspase 3, and caspase 8 in MDSCs derived from WT and MBL^-/-^ mice. *p<0.05. Data from one representative experiment of three independent experiments are presented.

TNF-α is a pleiotropic cytokine that signals through TNFR1 and TNFR2. The binding of TNF-α to the TNFR1 can activate the caspase-dependent cell death. TNFR1 also stimulates the formation of a cytoplasmic TRADD complex containing FADD and pro-caspase 8, leading to the activation of caspase 8 and initiation of an apoptotic signaling cascade. TNF-α binding to TNFR2 usually initiates immune modulation and tissue regeneration. To further evaluate the effect of TNF-α on MBL-mediated regulation of MDSCs survival, bone marrow cells were pre-treated with TNFR1 inhibitor, R-7050, before GM-CSF and IL-6 treatment in the presence of TNF-α. The frequency of MDSCs, G-MDSCs, and M-MDSCs was comparable between GM-CSF and IL-6 induced MDSCs differentiation with or without MBL upon TNFR1 blockade **(Fig. 8A, B, C)**. Similarly, the cell viability of MDSCs, G-MDSCs, and M-MDSCs had no difference between WT and MBL^-/-^ derived in the presence of R-7050 **(Fig. 8D, E, F)**. As shown in **Fig. 8G and 8H,** the levels of apoptosis-related molecules were comparable between MDSCs survival with or without MBL upon TNFR1 blockade. These results suggest that the regulation by MBL of MDSCs survival is mainly dependent on the TNF-α/TNFR1 signaling pathway.

## Discussion

MBL, which belongs to the collectin family of C-type lectins, is originally described to activate the complement system and defend against infection. In the present study, we demonstrated that MBL deficiency attenuated MDSCs differentiation by promoting cell apoptosis in the presence of TNF-α. In addition, we proposed that MBL actives TRADD/FADD/caspase3/8 pathway, leading to the inhibition of MDSCs viability. This, to our knowledge, is the first report to show that MBL acts on MDSCs survival under inflammation conditions, which may provide new insight into the mechanism of MBL in the MDSCs-related inflammatory diseases.

MDSCs are heterogeneous populations of immature myeloid progenitor cells with immunoregulatory functions. MDSCs participate in several autoimmune diseases, especially in rheumatoid arthritis. In addition, Th1 18, Th2 19, Th17 20, B cells 21, macrophages 22, mast cells 23, natural killer cells 24 and dendritic cells 25 involved in the progress of arthritis. Also, MDSCs can suppress B cells through NO and PGE2 26, promote Tregs proliferation through TGF-β 27, inhibit dendritic cells by reducing MHC-II and CD86 expression 28, and play immunosuppressive function by inhibiting T cells function. A plethora of inflammatory factors can stimulate the activation and proliferation of MDSCs, presenting in either bone marrow, spleen, or inflamed tissues. Studies have shown that several different factors, especially cytokines, influence the expansion and activation of MDSCs. Previous studies determined that IL-6-induced SOCS3 dysfunction and sustained activation of the JAK/STAT signaling pathway promoted the amplification and immunosuppressive function of breast cancer MDSCs in vitro and *in vivo* 29. Weber R 30 *et al.,* also reported that IL-6 induced CCR5 expression and strong immunosuppressive activity of MDSCs. Isatou Baha 31 *et al.,* support that IL-10 drives the molecular path that generates MDSCs and enhances immunosuppression during late sepsis. Another study defined IL-10 as a fundamental modulator of MDSCs within the ovarian tumor microenvironment, blockade of the IL-10 signaling network results in the alleviation of MDSCs-mediated immunosuppression 32. MDSCs from IL-10^-/-^ mice had increased phosphorylated signal transducer and activator of transcription 3 (p-STAT3) levels 33. It was reported that early treatment with LPS and IFN-γ could shift the differentiation of bone marrow progenitor cells (under GM-CSF conditions) from DC to MDSCs 34. Additionally, TNF-α, a vital cytokine involved in systemic inflammation, plays an important role in the differentiation and immunosuppressive function of MDSCs. TNF-α exhibited a dual function during chronic inflammation: arresting differentiation of immature MDSCs primarily via the S100A8 and S100A9 inflammatory proteins and their corresponding receptor (RAGE) and augmenting MDSCs suppressive activity^35^. TNF-α-mediated microRNA-136 induces differentiation of myeloid cells by targeting NFIA 36. Endogenous chronic TNF-α signaling promotes tumor growth by maintaining MDSCs survival in the transplanted tumor model 37. Leslie *et al.,* provides new insights into the crucial role of the TNF-α/TNFR2 pathway in MDSCs suppressive activity required during acute pleural infection to attenuate excessive inflammation generated by the infection 38. Other cytokines such as IL-6 can also activate the STAT3-DNMT axis silences the TNF-RIP1 necroptosis pathway to sustain survival and accumulation of MDSCs 39. Expression of TNF-α also attenuated Th1 cell-mediated inflammatory responses generated by the acute pleural mycobacterial infection in association with effective MDSCs expressing TNF-α and interacting with CD4^+^ T cells expressing TNFR2^38^.

MBL, an important part of the complement system, is mainly produced by the liver, and it can also be expressed by myeloid cells. Indeed, our and others' previous reports demonstrated that MBL could modulate innate immune cells' differentiation and function (DCs, monocytes, T cells, and mesenchymal cells) in different contexts. Here, we observed the adjuvant-treated MBL^-/-^ mice exhibited higher levels of TNF-α accompanied by a lower frequency of MDSCs compared with WT counterparts, indicating that MBL may boost the differentiation of MDSCs. Unexpectedly, MBL did not influence the ratio of MDSCs of mice under physiological conditions. Also, the immunosuppressive function of MDSCs were similar in MDSCs derived from WT and MBL^-/-^ mice. However, MBL^-/-^ mice exhibit decreased MDSCs of spleen and blood compared with WT mice after arthritis establishment. In arthritis, several cytokines, such as IL-1, IL-6, IL-8, IL-12, IL-17, TNF-α, and IFN-γ, are involved in almost all aspects of articular inflammation and destruction. We also observed that aggravated joint damage in MBL-/- mice was accompanied by a significant elevation of pro-inflammatory cytokines (IL-1β, TNF-α, IFN-γ and IL-6) and reduction of anti-inflammatory cytokine IL-10 in the serum compared to WT mice (Figure 2D). Agents targeting IL-6 such as tocilizumab attracted significant attention as a promising agent in RA treatment 40. IFN-γ^41^ and IL-1β^42^ are also potential targets for the treatment of rheumatoid arthritis. Furthermore, TNF-α has been considered a pivotal cytokine in the pathogenesis of RA, as significant clinical and laboratory evidence has been obtained by TNF-α blockade 43. The TNFR inhibitors represent the first rationally-based treatment and the first FDA-approved recombinant proteins for the treatment of RA 44. Since TNF-α is involved in the differentiation of MDSCs and the pathological process of arthritis, we speculate that MBL may be involved in the process of MDSCs differentiation caused by TNF-α. It is worth mentioning that the absence of MBL diminished the expansion and immunosuppressive effect of MDSCs upon TNF-α stimulation in vivo as excepted. Importantly, FADD, TRADD, caspase 3, and caspase 8 have been crucial for cell apoptosis caused by TNF-α-TNFR1 45. In the present study, we investigated the role of the apoptosis signaling pathway in MDSCs in the presence of TNF-α, and have demonstrated that MBL has an enhanced effect on MDSCs differentiation via downregulation of the FADD/TRADD/caspase 3/caspase 8 signaling pathways. In addition, the further results of TNFR1 inhibitor treatment indicate that MBL affects MDSCs survival through TNFR1.

## Conclusion

In summary, we demonstrate that MBL deficiency exacerbates inflammation through decreasing MDSCs accumulation partially. Importantly, our work elucidates an unknown feature of MBL function in MDSCs survival in the presence of TNF-α, indicating the association of MBL with MDSCs-related disease, especially in diseases with high TNF-α levels.

## Supplementary Material

Supplementary figure.

## Figures and Tables

**Figure 9:**
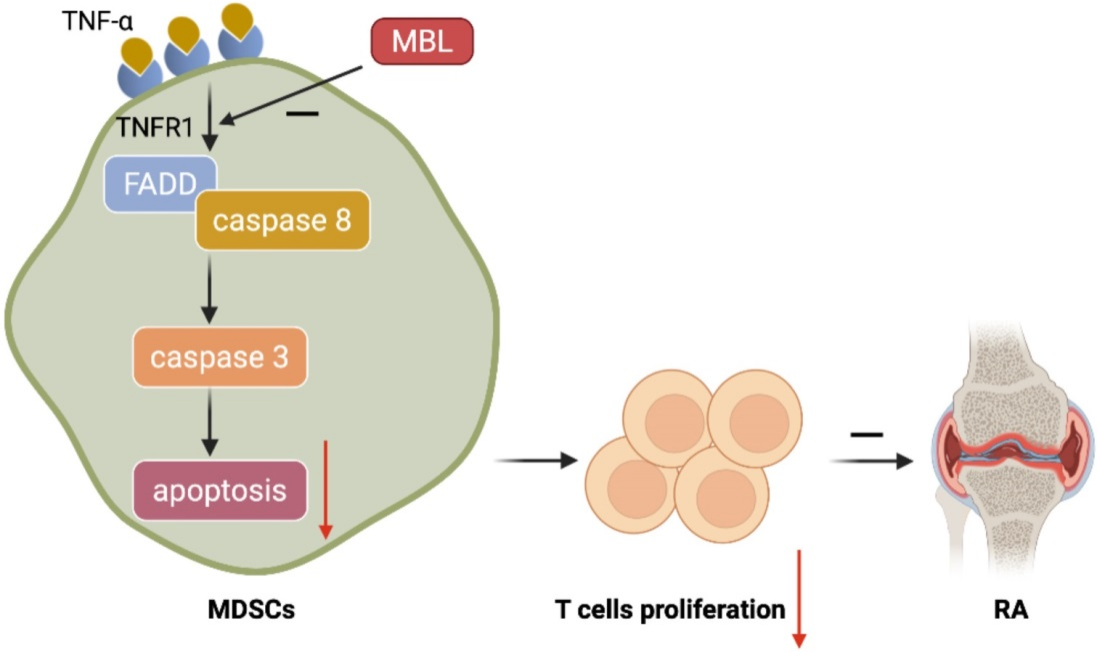
** Schematic representation of the mechanism by which MBL alleviates the progression of RA.** MBL inhibits the progression of mouse AIA via increasing the MDSCs frequency and decreasing T cells proliferation. Mechanistic studies established that MBL inhibited MDSCs apoptosis via down-regulating TNF-α/TNFR1-induced activation of FADD/caspase 8/caspase 3 signaling pathway.

**Table 1:** List of primer sequences used for RT-PCR analysis in this study.

	Forward primer (5′-3′)	Reverse primer (5′-3′)
Arg1	CAGAAGAATGGAAGAGTCAG	CAGATATGCAGGGAGTCACC
IL-10	CAAACAGTACGGAAACTCAACCT	GGTGATACAGATCCAGGGTGAAC
iNOS	ATGGACCAGTATAAGGCAAGC	GCTCTGGATGAGCCTATATTG
NOX2	ACTCCTTGGAGCACTGG	GTTCCTGTCCAGTTGTCTTCG
S100a8	AAATCACCATGCCCTCTACAAG	CCCACTTTTATCACCATCGCAA
GAPDH	TTGTCATGGGAGTGAACGAGA	CAGGCAGTTGGTGGTACAGG
