# Supplementary figures and images for: Mannan-Binding Lectin Deficiency Limits Inflammation-induced Myeloid-Derived Suppressor Cells Expansion via Modulating Tumor Necrosis Factor Alpha-triggered Apoptosis
Source: Int J Biol Sci. 2022 Jan 26;18(4):1580–93. doi: 10.7150/ijbs.68865 (PMC8898356; doi:10.7150/ijbs.68865)

Sup. Figure 1

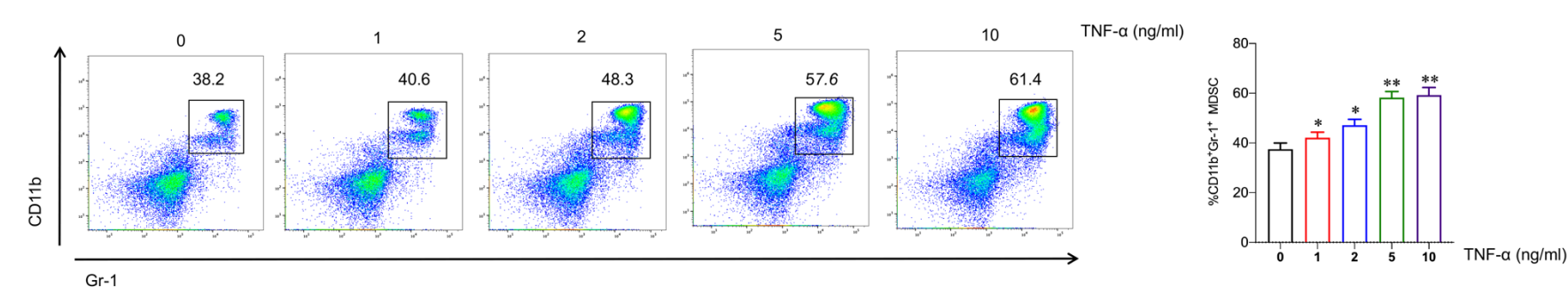

Supplement: Supplementary file 1 — Supplementary figure. [file ijbsv18p1580s1.pdf]
